# The evolution of social norms interventions for health promotion: Distinguishing norms correction and norms transformation

**DOI:** 10.7189/jogh.11.03065

**Published:** 2021-07-10

**Authors:** Beniamino Cislaghi, Alan D Berkowitz

**Affiliations:** 1 London School of Hygiene and Tropical Medicine, London, UK; 2 Independent consultant, Mount Shasta, California, USA

The evidence that social norms – informal rules of acceptable action within a given group – influence people’s health-related choices is abundant and cross-sectorial, ranging from health-related behaviours such use of contraception, handwashing, alcohol use and/or smoking, to social practices and behaviors such as corporal punishment, child marriage, sexual harassment and sexual assault [1]. Following the large body of literature, especially coming in health promotion, underscoring the importance of addressing harmful social norms to improve people’s health, social norms theory has long been used to inform public health policy and practice [2].

Even though several different theoretical perspectives exist on what social norms are and how they affect people’s practices [1], much contemporary research and practice in public and global health has adopted theory and terminology by Cialdini and colleagues [3], whose Focus Theory of Normative Conduct defined social norms as (1) one’s beliefs about what others in one’s group do (*descriptive norms)*, and (2) the extent to which one believes others as approving or disapproving of something (*injunctive norms)*.

## FROM NORMS CORRECTION TO NORMS TRANSFORMATION

### The original approach: the *norms correction* strategy

Drawing on Cialdini’s theory of how social norms affect behaviour and how they are defined, a stream of health promotion practice emerged in global and public health that aimed at addressing harmful social norms. This first stream of work we mentioned has traditionally been referred to as the “social norms approach”, but it has also been referred to as “the perceived norms model”, “social norming”, the “norms challenging model” or – our preferred terminology – the norm correction strategy (NCS) [4]. Initiated in the mid-1980s by Perkins and Berkowitz [5], the development of the NCS followed two findings, respectively on 1) the importance of peer influence can play on people’s health-related choices and actions and 2) the fact that healthy attitudes and behaviours tend to be underestimated, while unhealthy attitudes and behaviours tend to be overestimated [4]. Initial empirical studies on social norms documented that people’s normative beliefs were inaccurate: their beliefs did not correspond to what others actually did and approved of. The NCS was proven effective in reducing a variety of harmful health-related behaviours, such as, for instance: alcohol use, tobacco use, sexual assault and drink-driving. NCS most commonly conveyed the message about people’s behaviours and approval in social marketing media campaigns, but some NCS interventions also did so in small group workshops in which the group norm is assessed and the misperception is discussed.

### An emerging approach: the *norms transformation* strategy

At the beginning of the 2000s, interventions that targeted social norms sustaining the practice of female genital cutting (FGC) in West Africa offered an important contribution to addressing harmful practices in rural West African villages. Researchers who studied the three-year community-led programme implemented in rural Senegal by the NGO Tostan suggested this model was effective in reducing FGC because it addressed existing social norms that were perceived as strongly sustaining the practice [6]. Yet, their Community Empowerment Programme (CEP) – an example of what we called “norms transformation strategies” (NTS) – was different from the norm correction strategy described above in two regards.

**Figure:**
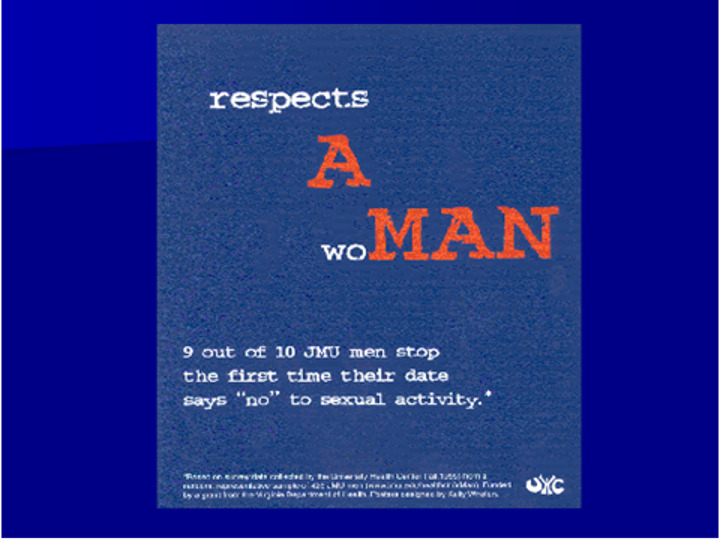
Photo: Fostering Healthy Norms to Reduce Violence in our Communities: A Social Norms Toolkit (source: Alan Berkowitz, used with permission).

First, the CEP did not only focus on social norms: it addressed a variety of intersecting institutional, material, individual and social factors that sustained them. Second, the CEP didn’t try to correct misperceptions. It was instead to focus on changing people’s attitudes, behaviours and, eventually, social norms. Their programme begins with a core group of 50 people participating in a series of facilitated conversations. This group of people eventually becomes large and confident enough to publicly enact new positive practices that challenge the existing community norms. This larger shift in what people did and said to approve created a new positive norm that motivated compliance even in the most sceptical onlookers. Later, the literature gave to this process (through which group discussions expand to include a larger number of community members eventually challenging social norms) the name “organized diffusion” [7].

## PRACTICAL IMPLICATIONS IN CHOOSING THE MOST APPROPRIATE STRATEGY

We identified four characteristics of the context that can be helpful in the formative phase of intervention design: 1) the norm/reality relation, 2) the heterogeneity of the group, 3) the normative constellation around the harmful practice, and 4) the role played by the larger ecology of non-normative factors (**Table 1**).

**Table 1:** Summary of practical consideration for choosing NCS or NTS

Contextual characteristics	Norms correction strategy are more appropriate when	Norms transformation strategy are more appropriate when
Norm/Reality Relation	Norms are a misrepresentation of reality	Norms are a correct representation of reality
Heterogeneity of the group	The group is fairly homogeneous	The group is fairly heterogeneous
The constellation of norms	One direct norm sustains the practice	One or several indirect norms sustain the practice
The role of other factors	Correcting the misperceived norm can achieve change	Other factors contribute to the practice as much as the norm

NCS – norms correction strategy, NTS – norms transformation strategy

The relation between norms and peoples’ true actions and attitudes is the first characteristic to take into account. If everyone in a given group disapproves of child marriage, but believes everyone else approves of it, the misalignment between the injunctive norm and people’s attitudes is large. The larger the misalignment between normative beliefs and reality, the greater the scope to adopt an NCS design that uncovers that gap. That could be achieved with an intervention unveiling that gap, for instance through posters about people’s true attitudes in the community. Conversely, NTS approaches might be more appropriate when norms are correct or nearly correct representations of other people’s behaviours and attitudes (ie, there is no misperception or the misperception is small) or when the norm is so strongly held that it wouldn’t be credible to suggest it is misperceived. In these cases, NTS can be effective in facilitating the gathering of a core group of change actors with protective attitudes and that expands over time.

Heterogeneity (group composition) and size of the group should also be taken into account when designing a social norms intervention. Recent social norms research has pointed to the importance of highlighting similarities between the group portrayed in an NCS campaign and the receivers of that campaign. We hence suggest that the less heterogenous a group is, the greater the potential of using an NCS approach to correct norms within that group. Conversely, the more heterogenous the group is, the more an NTS approach might be appropriate, as it would help create cross-group dialogue and cultural cross-fertilisation. It might be possible to combine the approaches: the NTS could be used nationally within different ethnic and language groups, while a complementary NCS media campaign could be developed that is group specific, creating synergy between the groups.

Cislaghi and Heise [8] have suggested that norms can have direct and indirect influence. They have direct influence when the practice is in itself normative. For instance, when the practice of drinking is sustained by an injunctive norm that “students approve of those who get drunk on Saturday night”. Norms can have indirect influence when the practice is not normative but there exists a system of norms that contributes to keeping the practice in place. When norms have direct influence, NCS might be more appropriate, as they target directly a norm that sustains the practice for the group that is salient to the behaviour. When the constellation of norms is slightly more far removed from the actual practice, NTS seem a better choice, as they allow time to participants to identify that system of norms and discuss opportunities and strategies to address them.

Finally, we suggest that it’s important to understand how norms interlock with other factors in sustaining the harmful practices of interest. Since they focus exclusively on correcting norms, NCS are more appropriate when the practice of interest is driven by a social norm targeted by the intervention or when practitioners are intentionally mostly concerned with correcting that specific norm, accounting for possible beneficial spill-over effects. NTS are instead more appropriate when the constellation of intersecting material, institutional, individual and social contribute equally in sustaining the harmful practice [9]. For instance, NTS can be implemented through a series of participant-led conversations (workshops for reference groups) that result in the design of culturally-compatible change strategies. NTS create space for people to devise socio-political solutions that are appropriate to their context, as they experience it in its real-life complexities. One such key factor, for instance, are power relations between members of the same group, frequently missing from social norms work, but more present in the gender norms literature, specifically [10].

## ETHICAL REFLECTIONS ON CHANGING SOCIAL NORMS

Policy-makers and development practitioners tend to be thirsty for knowledge on how to change social norms but are less eager to engage in discussions on the moral justification to do so and on the most ethical approaches. Yet, both kinds of social norms interventions present important ethical dilemmas that require purposeful thought and action. Here, we look at three key points of ethical tension that arise when implementing social norms interventions, summarised in **Table 2****.** We do not mean for these to be exhaustive; rather, we wish to begin the conversation across donors, researchers, and practitioners on what ethical social norms interventions look like.

**Table 2:** Ethical tension points arising when using NCS and NTS for health promotion

Ethical tension point	Norms correction strategies			Norms transformation strategies	
	**Risks**	**Moderation**	**Risks**		**Moderation**	
1. Agenda Setting: Who decides what norm to target?	High risk. Outsiders might target a norm that has hidden prosocial functions obtaining harmful intervention outcomes.	Engage community members at design stage in identification of key norm to change.	Medium risk. Outsiders might target the ‘wrong’ norm, but participants could alert practitioners and redirect their efforts as the intervention unfolds.		Work with community members as process leaders and decision-makers throughout the intervention and its evaluation.	
2. Social Engineering: Who decides the solution to the problem?	Low risk. The project tells the community about itself, not what to do with this knowledge.	Design media campaigns where the data source is clearly showed, so that its credibility can be assessed by the audience.	High risk. Potentially a community facilitator could orient the community in the direction she or he wants them to go.		Design critical curriculum where the authority is in the process, not in the facilitator. Train facilitators to help communities achieve their own goals.	
3. Harmful Effects: Who protects the compliers with the old norm from ostracization?	High Risk. The project stops once the message is communicated.	Include neutral messages that do not ostracize compliers	Medium Risk. A possibility exists that community members might actively police compliers with the old norms.		Design interventions that include space for slow-paced mutual understanding and conciliation.	

The first point refers to who decides what social norm should be addressed by the intervention. Advocates of community participation in project design and implementation have long argued that future participants should be co-designers and co-implementers of the intervention both for ethical and effectiveness purposes, an approach that has also been shown to result in more successful interventions [11]. Since they are mostly carried out as media campaigns, NCS could engage participants at the issue identification and campaign design phases. NTS instead, in the model we proposed above, can course-correct their strategy and goals to match participants’ evolving objectives as they begin to implement the norms transformation strategy.

The second, related point is on who decides what positive norms the community should adopt. NCS do not try to resolve contradictions and power relations within the community but tell the community about itself. NTS, instead, aims to achieve improvements in people’s lives by holding conversations with transformative potential that facilitate change not only in people’s normative beliefs, but also in their attitudes and actions. As such, NTS are open to the criticisms of social engineering non-culturally familiar societies from the outside-in. Yet, NTS can be effective in addressing global health and social justice issues, evidence shows that, for instance, it can have a good impact in reducing child marriage or domestic violence [12]. A potential solution to the conundrum could draw from the Freire’s progressive approach to education [13]. In his model, practitioners become facilitators of a community process where participants uncover their individual and collective aspirations and eventually co-design a process to achieve them. This model is today constrained by the mainstreaming development funding model that is based on the achievement of donor-selected results as measures of success.

The third and last ethical point of tension relates to the harmful consequences of interventions that address social norms. Even when they achieve a positive impact flipping a harmful norm, the few remaining compliers may be exposed to ostracization, especially when the campaign makes the mistake of bearing language that portrays norm compliers negatively. For this reason, NCS often include neutral messages, that refrain from suggesting that people are ‘good’ or ‘bad’ because they comply (or don’t) with a given norm. Even when the message is framed positively, ethical implications emerge if the intervention promotes as normative an action that some sectors of the population are unable to perform. While one person might be able to change some behaviours independently of their existing material or institutional context (eg, refraining from harassing sexually someone), adopting other positive behaviours might depend on some material or institutional resources unavailable to the most vulnerable populations. Both NCS and NTS face this risk when they do not tackle other factors that might make it impossible for some to comply with the new positive norm. NCS would need to phrase its messages to raise compassion, rather than anger or disappointment, towards the compliers with the harmful norm. And, similarly, NTS should create a space where participants can find conciliatory ways to overcome the wrong/right action divide and reframe the behavioural change narrative into one of mutual and collective care, responsibilities, and trust.

While designing effective interventions is considerably important, we hope that practitioners will pair reflections on effectiveness with purposeful ethical action. We offer our key reflections on what can help interventions that integrate social norms component be both more effective and ethical, in the hope that the development industry will increasingly be also interested in the latter, for a world where people are not only concerned with interventions that work, but where they also care about knowing they are doing the right thing.
